# Pangenome analysis of nine soybean cyst nematode genomes reveals hidden variation contributing to diversity and adaptation

**DOI:** 10.1186/s12864-025-12493-x

**Published:** 2026-01-15

**Authors:** Lucas Borges dos Santos, Kurt C. Showmaker, Rick E. Masonbrink, Kimberly K.O. Walden, João P. Gomes Viana, Khee-Man Kwon, Alvaro G. Hernandez, Zhihai Zhang, Christopher J. Fields, Thomas R. Maier, Andrew J. Severin, Thomas J. Baum, Melissa G. Mitchum, Matthew Hudson

**Affiliations:** 1 Department of Crop Sciences, University of Illinois at Urbana-Champaign, Urbana, IL 61801 USA; 2 Dasomics LLC, Madison, MS USA; 3 Genome Informatics Facility, Iowa State University, Ames, IA 50011 USA; 4 Roy J. Carver Biotechnology Center, University of Illinois at Urbana-Champaign, Urbana, IL 61801 USA; 5 National Center for Supercomputing Applications, University of Illinois at Urbana-Champaign, Urbana, IL 61801 USA; 6 Department of Plant Pathology, University of Wisconsin-Madison, Madison, WI 53706 USA; 7 DOE Center for Advanced Bioenergy and Bioproducts Innovation (CABBI), University of Illinois at Urbana-Champaign, Urbana, IL 61801 USA; 8 Department of Plant Pathology, Entomology and Microbiology, Iowa State University, Ames, IA 50011 USA; 9 Department of Plant Pathology and Institute of Plant Breeding, Genetics and Genomics, University of Georgia, Athens, GA 30602 USA

**Keywords:** Soybean cyst nematode, Pangenome, Genome evolution

## Abstract

**Background:**

The soybean cyst nematode (SCN) is a persistent threat to soybean production. SCN populations continually overcome resistant cultivars, causing significant yield losses. Studies conducted with a single reference genome restrict our understanding of intraspecific diversity, masking significant mechanisms of virulence evolution and host adaptation. Here we report a pangenome constructed of nine SCN populations of different pathotypes, including eight newly generated high-fidelity genome assemblies.

**Results:**

We detected over 19,000 orthologous gene families and more than 12,000 putative secreted proteins in SCN. Combined, these data indicate substantial diversity across populations. Gene content analysis showed that 35% of gene families were the conserved core, 15% were soft-core, and 48% were accessory. Evidence of rapid evolution was identified in a high portion (40%) of core single-copy genes, most notably inside the protein domains responsible for host recognition and immune modulation. Analysis of gene-family expansion revealed extensive duplication and loss across lineages, suggesting ongoing paralog turnover within SCN populations. Finally, a graph-based pangenome enabled the identification of numerous structural variants within regions under selection.

**Conclusions:**

Our study highlights substantial genetic variation in SCN that is not captured by single-reference analyses. By integrating multiple high-quality assemblies, we show that the SCN genome is highly dynamic, with extensive gene duplication and loss as well as structural variation shaping the differences among nematode populations. Collectively, the SCN pangenome provides a robust resource for studying virulence and adaptation mechanisms in SCN and establishes a genomic foundation for the development of more precise management strategies.

**Supplementary Information:**

The online version contains supplementary material available at 10.1186/s12864-025-12493-x.

## Introduction

The soybean cyst nematode (SCN), *Heterodera glycines*, is the most damaging pathogen of soybean, responsible for annual losses of approximately $1.5 billion in the United States alone [1–3]. This microscopic, parasitic nematode penetrates the soybean roots to establish feeding cells, leading to stunted growth and reduced crop yield. One of the major hurdles in managing SCN is its persistence in soil, making complete eradication unfeasible. Management strategies like crop rotation and SCN-resistant soybean varieties are designed to suppress population densities, rather than completely eradicate nematode populations. However, it has been observed that SCN populations can rapidly adapt to overcome these measures [4–7], highlighting the need for continued innovation for more effective and durable management approaches.

Recent advances in genomics have begun to uncover the complex nature of the SCN genome. Preliminary studies have indicated that SCN may be genetically diverse, partly due to its sexual mode of reproduction, which enables extensive genetic recombination and contributes to its ability to adapt quickly to the available sources of resistance [8, 9]. The two publicly available chromosomal-level genome assemblies of SCN highlight the genomic architecture and genetic repertoire for the species [10, 11]. A more recent study comparing two independently derived population pairs of SCN identified two genomic regions showing signatures of differential selection for virulence on different soybean genotypes [12].

SCN virulence mechanisms involve the secretion of effector proteins that manipulate host plant cell structure and function to facilitate infection and feeding [13]. In many plant-pathogen systems, resistance (R) genes detect specific pathogen effectors and activate defense responses through effector-triggered immunity (ETI) [14]. This dynamic interplay between pathogens’ effector proteins and their hosts’ resistance genes characterizes a co-evolutionary arms race [15], in which the pathogen often maintains an advantage over the host due to larger population sizes and shorter generation times. In the case of SCN resistance genes (also known as *Rhg* genes), resistance mechanisms appear more complicated than the classic effector-receptor paradigm, with vesicular trafficking and hormone systems being implicated, as well as 1-C folate metabolism [16–20], but the evolutionary scenario is very likely to be similar. This scenario introduces selective pressures that continually shape the evolution of virulence effectors [21].

Understanding intra-specific variation is essential for comprehending intricate aspects of adaptation. Advances in sequencing technologies have enabled the generation of highly contiguous genome assemblies. In SCN, there are persistent challenges in isolating sufficient high-quality genomic material from single nematodes for long-read sequencing, which leads to the reliance on pooled individuals to generate whole-genome assemblies, limiting the resolution of intra-population variability. The initial SCN reference genome, of the TN10 line, revealed important aspects of the genome architecture of the species [22]. While informative, a single composite reference genome may fail to capture the full extent of genetic variation in species with extensive hypervariability, leading to reference bias due to variants absent from the assembly [23]. This limitation becomes especially relevant when comparing SCN subspecies or pathotypes – known as *Heterodera glycines* (HG) types – based on an established system to categorize SCN populations according to their virulence profiles on a set of soybean indicator lines [24, 25].

The advent of pangenomes as reference systems provides a powerful solution to study the genetic diversity among diverged populations. Despite multiple definitions, here the pangenome is defined as the incorporation of multiple high-quality genomes into the analysis workflow, allowing for a more comprehensive representation of the genetic landscape within a given species or clade [26, 27]. Pangenomes are typically described as “open” or “closed,” depending on whether new genes continue to accumulate as more genomes are added. A closed pangenome suggests a largely finite gene repertoire, while an open pangenome reflects ongoing gene gain and high genomic plasticity, with implications for adaptation and evolutionary potential [28]. The alignment of complete genomic sequences into variation graphs provides a robust framework for detecting presence-absence variations (PAVs) and other types of structural variants, facilitating the search for selection sweeps and regions associated with traits of interest [27, 29].

In this study, we present the first comprehensive genomic characterization of intra-specific diversity in SCN using a graph-based pangenomic framework. We report high-quality reference assemblies for seven new populations of SCN and expand the assessment of two previously published [10, 11]. We hypothesize that the SCN pangenome can more accurately unveil the comprehensive genetic variation associated with different HG types, crucial for understanding SCN’s adaptability and virulence. Comparative analyses shed light on core (i.e. shared) and accessory/dispensable genes (lineage specific), revealing significant PAVs and signs of more rapid evolution that underpin potential targets for future management strategies of SCN. This study not only enriches our understanding of SCN’s genetic complexity but also provides powerful resources for future research in SCN, underscoring the potential of pangenomic approaches in revealing the intricate dynamics of pathogen-host interactions.

## Results

### Chromosome-level assemblies of 9 SCN genomes

**Fig. 1:**
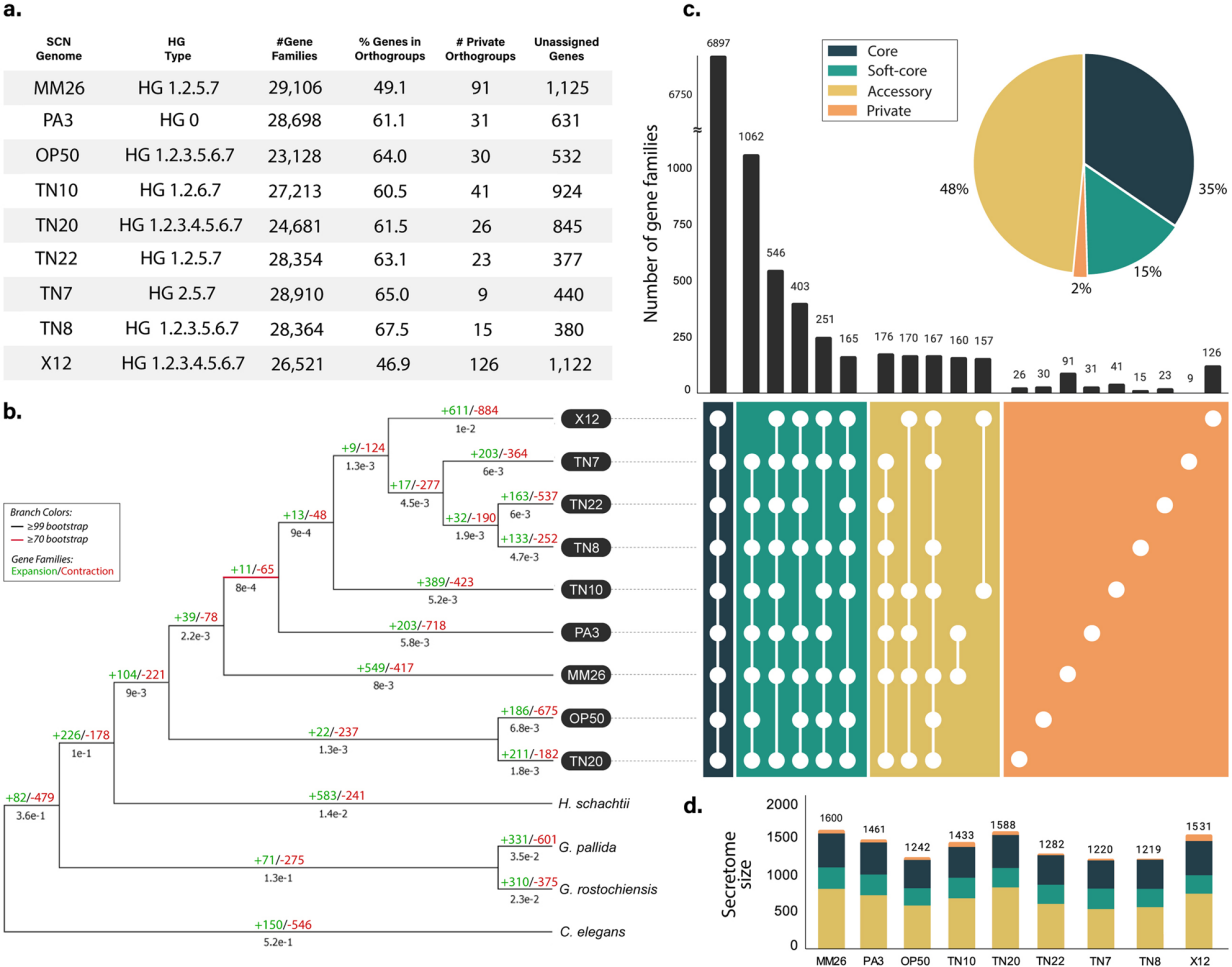
The landscape of the SCN pangenome. **a** Descriptive statistics of the ortholog analysis in SCN and their HG types. **b** Phylogenetic relationships among nine SCN genomes (TN7, TN8, etc.) and four outgroup species, based on 803 single-copy ortholog genes shared among them. For each branch, gene family contraction and expansion events are marked in red and green, respectively. SCN genomes are aligned with the figure on the right. **c** The number of orthogroups (i.e.: gene families) shared among the core, soft-core, accessory and private genomes. For each combination (black), the number of genomes included is color-coded in the UpSet plot, and the absolute number of shared gene families is in black. For the soft-core and accessory, only the top 5 combinations are displayed. **d** The secretome size (total number of secreted proteins) across the SCN genomes

We analyzed nine representative populations of *Heterodera glycines* (SCN) spanning various HG types (Fig. 1a). A genome assembly for the line X12 [10] was obtained from publicly available datasets, while eight populations (MM26, OP50, PA3, TN7, TN8, TN10, TN20, and TN22) were sequenced using a combination of multiple high-throughput sequencing technologies to enable chromosome-level resolution for the pangenome construction. In the case of TN10, the existing reference genome assembly and annotation was improved to match the other new genomes.

Six populations (TN7, TN8, TN20, TN22, OP50, and MM26) were sequenced using Oxford Nanopore Technologies (ONT). This approach produced long-read datasets with depths ranging from 30× to 74× (Supplementary Table S2). For MM26 and PA3, we employed PacBio Sequel II with HiFi chemistry, generating high-fidelity long reads with depths of 89× and 88×, respectively. Additionally, PA3 was sequenced using PacBio continuous long reads (CLR), yielding 8.3 Gb (approximately 69× coverage). MM26 also received ultralong ONT sequencing, contributing an additional 9× depth.

To improve scaffolding and support chromosome-level assembly, we generated Illumina HiSeq paired-end reads and applied Dovetail’s proximity ligation technologies (Hi-C and Chicago) across all seven newly sequenced populations. Illumina Hi-C coverage ranged from 73× to 88×, while Chicago coverage ranged from 58× to 87×. These data enabled the assembly of nine pseudo-chromosomes per genome, consistent with the haploid karyotype of SCN [30]. The assembled chromosomes spanned from 8.21 Mb to 20.83 Mb in length. Each assembly also included a set of unplaced scaffolds, ranging from 58 to 1,178 fragments, comprising 0.7% to 16.1% of total assembly size (Table 1).

We annotated protein-coding genes across the nine SCN genomes using a combination of RNA-seq and long-read cDNA sequencing of the TN10 population to improve transcript-based gene predictions. On average, each SCN genome contained approximately 19,889 gene models, with gene counts ranging from 16,287 to 24,795 (Table 1). The annotated genes displayed a high degree of completeness, as assessed by BUSCO analysis over the predicted proteins, with scores ranging between 96.3% and 93.8% for the new assemblies and 87.8% for X12 (Table 1, Supplementary Table S3).

Gene structure analysis revealed that SCN genes had an average coding sequence (CDS) length of 1.3 kb, while the average gene length was 3.4 kb. A subset of genes contained alternative splicing events, with an average of 68% of genes exhibiting at least one alternative transcript. To assign functional annotations, we compared SCN gene models against protein databases, leading to putative functional assignments for 69.11% of genes. Among these, genes associated with key population-specific biological processes, including parasitism-related functions, were identified. Comparative analysis of gene families revealed 109 expanded gene families in SCN relative to other nematodes, including those linked to plant-pathogen interactions.

The repeat-annotation analysis performed on the seven SCN genomes identified an average of 41.49% (51.85 Mb) of repeat sequences (Supplementary Table S3). The repetitive elements are mainly composed of interspersed repeats (37.54%), which include long terminal repeats (LTRs, 3.28%), long interspersed nuclear elements (LINEs, 0.88%), DNA transposons (4.02%), and short interspersed nuclear elements (SINEs, 0.03%). A substantial portion of the interspersed repeats (29.33%) remained unclassified. Simple repeats and satellite sequences accounted for 1.78% and 0.2% of the repetitive bases, respectively, and other types accounted for 1.97%.

### Genome Organization and Phylogeny of SCN

**Fig. 2:**
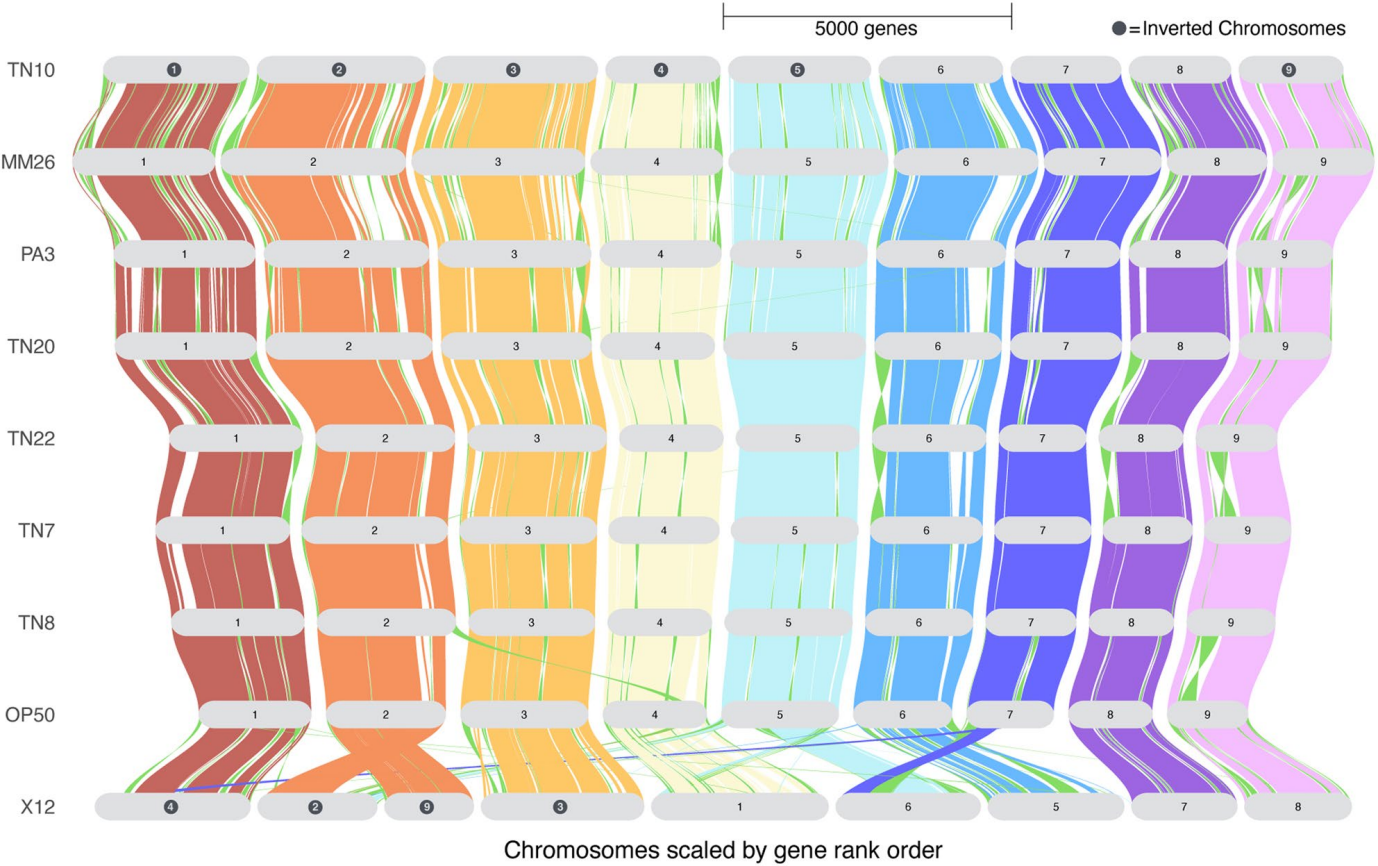
Syntenic map of orthologous regions across nine SCN genomes. Chromosomes are ordered and color-coded to maximize synteny with the reference (TN10). Inverted chromosomes are labeled with a black circle. Large structural inversions are highlighted in green

To study the structural differences across the SCN pangenome, we performed a synteny analysis highlighting the position of major syntenic regions across the nine SCN genomes (Fig. 2). The analysis indicates that the seven de novo genomes present high collinearity, with large blocks of orthologous and paralogous genes organized in a similar order. Genomic inversions appeared to be more pronounced around telomeric regions, and a few small translocations appeared in chromosomes 2, 4, and 6. The TN10 genome shows a high degree of structural similarity to the seven newly assembled genomes, while the previously released assembly of X12 [10], shows the lowest degree of synteny conservation and the highest number of structural misarrangements and gaps. Although some of these differences may reflect structural polymorphisms between populations, the extent and pattern of discordance observed in the X12 assembly suggest that many of the rearrangements are more likely due to sequencing and assembly limitations, including lower contiguity or misassembly in earlier-generation technologies. These findings underscore the value of high-quality, long-read assemblies for comparative genomic analyses and provide a refined view of chromosomal organization across SCN lineages.

To further elucidate the evolutionary relationships of SCN, we conducted a phylogenetic tree reconstruction including four additional species as outgroups: *H. schachtii*, *G. pallida*, *G. rostochiensis*, and *C. elegans*. We focused our analysis on genes of which we were able to recover the complete protein-coding sequences from every accession. In total, we utilized 843 single-copy orthologs shared between the 9 SCN genomes and the 4 outgroup species. The resulting tree (Fig. 1b) provides valuable insights into the phylogenetic relationships and evolutionary distances between different lineages of SCN and between SCN and other cyst nematode species of the genera *Heterodera* and *Globodera*, as well as the model organism *C. elegans*. The outgroup species are organized following the previously proposed nematode phylogeny [31], where the beet cyst nematode (*H. schachtii*) is placed as the nearest relative to SCN, followed by the potato cyst nematodes (*Globodera spp.* clade), and more distantly related to the free-living nematode and model organism, *C. elegans*. The SCN genomes clustered into a monophyletic group with high bootstrap support (> 99). While genomes derived from in vitro populations may not provide an accurate representation of clonal lineage structure due to potential heterogeneity and admixture, they offer valuable insights into broader phylogenetic trends and population-specific divergence.

Here, TN20 and OP50 formed a distinct sub-clade, and TN10 contained the branch with the lowest support value (70). Interestingly, TN10 also showed significant gene family flux (+ 389 expansions/−423 contractions). The next internal node grouped TN8, TN22, and TN7, which share relatively close relationships. X12 formed a separate, more basal lineage within SCN, positioned as an early diverging isolate in the group, which may be influenced by the assembly quality and synteny differences. PA3 and MM26 grouped with strong support and displayed the most extreme values in gene family dynamics within SCN. MM26 had the largest net expansion (+ 549/−417), while PA3 had one of the most pronounced contractions (+ 203/−718), indicating divergent evolutionary pressures or differences in selective environments, even within a shared lineage. These observations indicate a cohesive clade for SCN with considerable intra-specific diversity existing in both sequence divergence and gene family dynamics.

### Core and Accessory Genomes

**Fig. 3:**
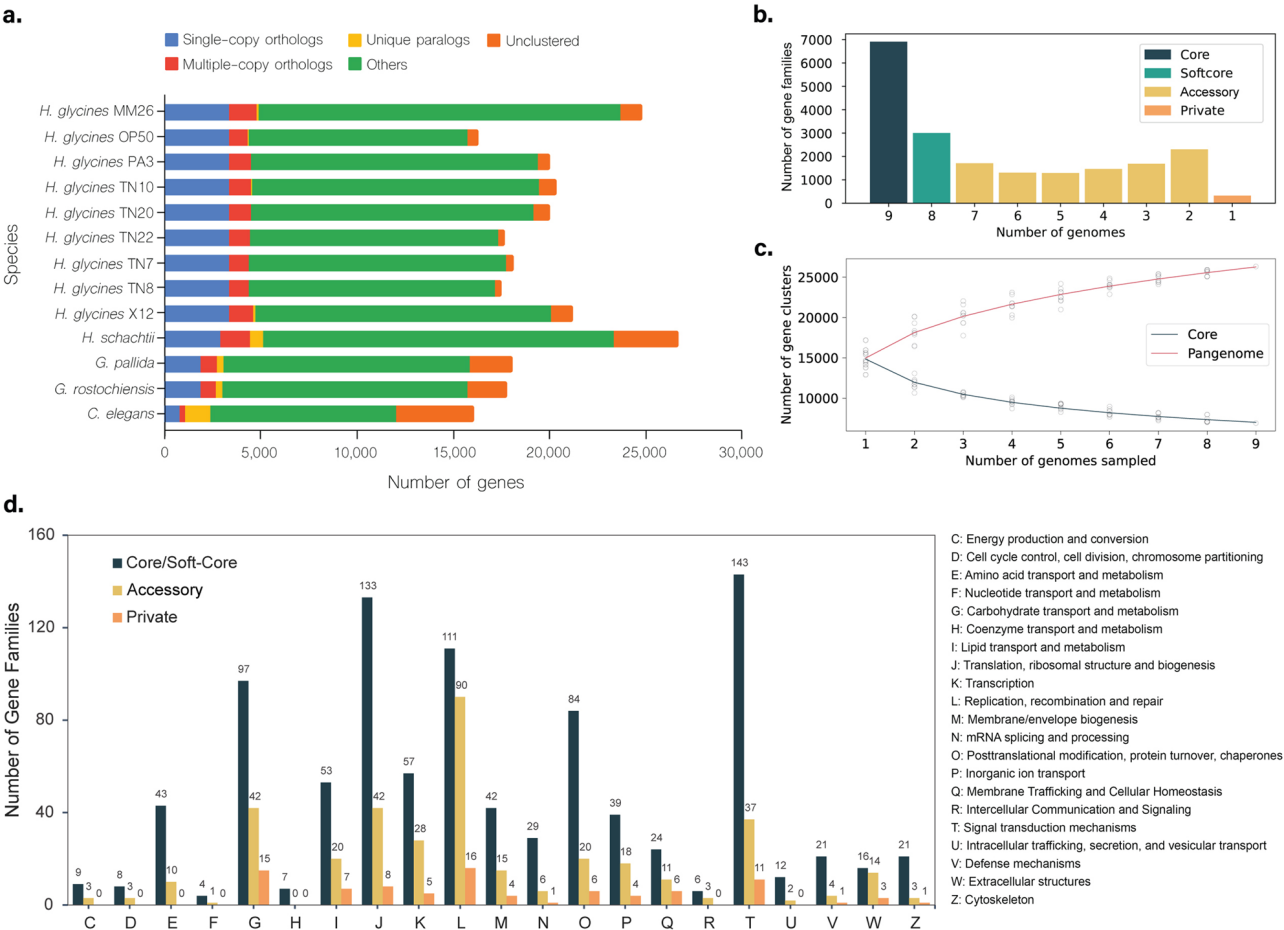
Characteristics of the SCN pangenome. **a** Gene family classification across *H. glycines* populations and related nematodes. Predicted genes were clustered into orthogroups using OrthoFinder and categorized as single-copy orthologs (present once in all species), multiple-copy orthologs (orthogroups with gene duplications), unique paralogs (species-specific paralogous genes), unclustered (genes not assigned to any orthogroup), and others. **b** Histogram of the cumulative number of ortholog gene families across nine genomes. **c** Core and pan-genome sizes of SCN. Scatter points represent the cumulative variation across nine genomes derived from different populations. **d** Functional annotation of the core/soft-core, accessory, and private SCN genomes. The top-scoring GO terms were grouped into 21 major categories from C-Z

To explore the genomic diversity and evolutionary dynamics across the genus *Heterodera*, we utilized a pangenome approach encompassing the diversity of nine SCN genomes. A total of 169,702 predicted peptide sequences of all nine SCN genomes were clustered into 19,951 gene families based on protein homology (Supplementary Table S4). Across species, most genes were classified as unique paralogs, while single-copy orthologs represented only a small fraction of the total gene repertoire (Fig. 3a), indicating extensive gene family expansion and diversification within SCN.

Based on orthogroup clustering, the core, soft-core, private and accessory SCN genomes were determined. The core genome (i.e. genes present in all nine genomes) is composed of 6,897 genes (34.6%); the soft-core genome (genes present in 8 out of nine genomes) consists of 2,990 genes (15%) (Supplementary Table S5), which represent nearly universal genes that may be absent in one genome due to assembly and annotation gaps. The accessory genome is defined as the group of genes present in 2 to 7 genomes, and the private genomes as genes present in only one genome. We identified 9,672 accessory genes (48.5%), and 392 (2%) private genes (Supplementary Table S6). The distribution of these genes across each number of genomes is shown in Fig. 3b.

To assess the openness of the SCN pangenome, we performed a rarefaction analysis by incrementally adding genomes and tracking the number of gene families recovered (Fig. 3c). The resulting curves indicate that neither the core nor the pangenome reached full stabilization with the nine genomes analyzed. While the core genome showed a rapid decline followed by a partial plateau, the pangenome continued to expand as additional genomes were incorporated, resembling the trajectory of an open pangenome model rather than a closed one [28]. These results suggest that a portion of genomic diversity may remain uncharacterized and that additional SCN genomes will likely yield new gene families not captured in the current dataset.

A gene ontology (GO) enrichment analysis of the core/soft-core and accessory genomes identified key biological pathways associated with them (Fig. 3 d). The core SCN genome is associated with fundamental biological functions, with nine out its ten most scoring GO categories associated with functions such as transcription, protein translation, DNA binding (Supplementary Tables S7, S8, S9). The accessory genome is enriched for functions such as biotic stimulus, cell wall modification and ion homeostasis (Supplementary Tables S7, S8, S9).

### Protein-Coding Selection Analysis

**Fig. 4:**
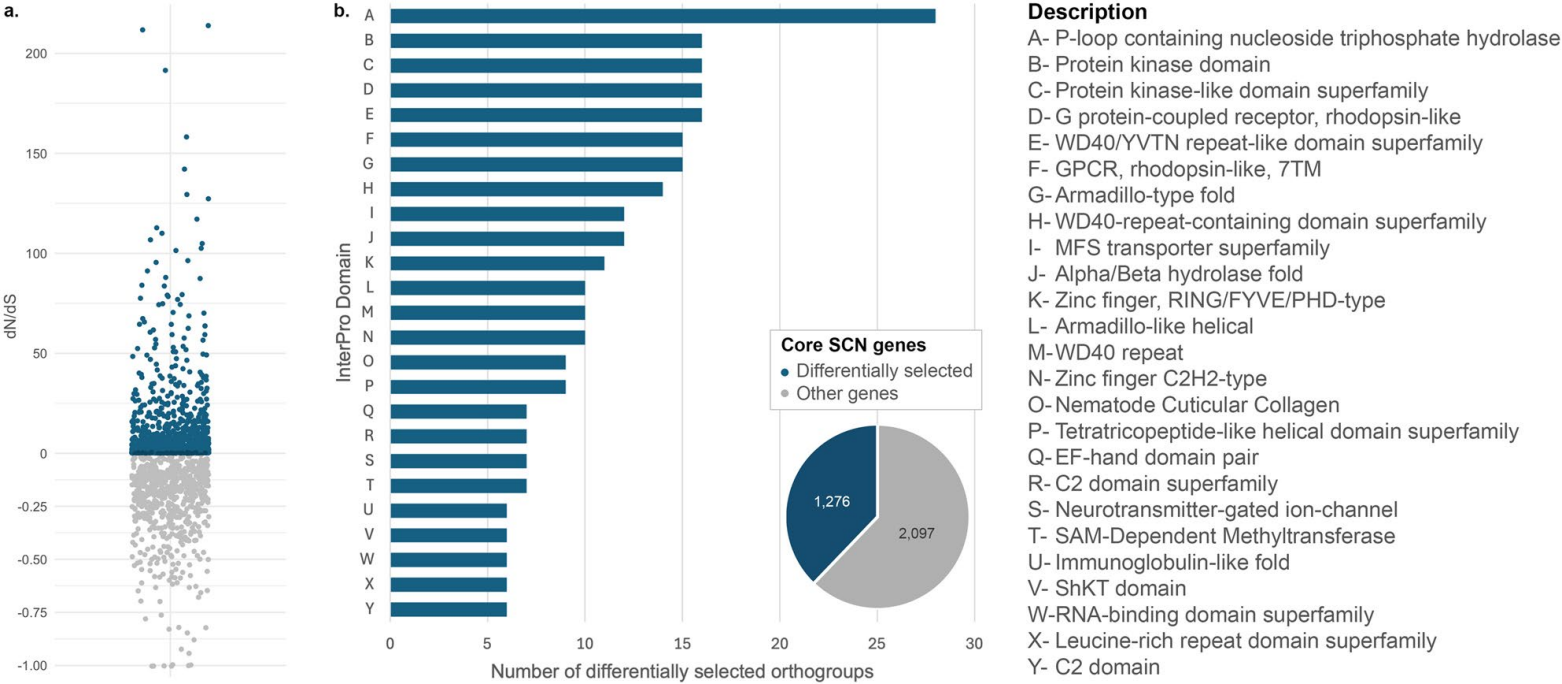
Peptide sequence level selection analysis. **a** Distribution of dN/dS among 2,881 core SCN genes. Genes presenting dN/dS > 1 were considered as rapidly evolving. **b** Domain annotation of 2,881 core SCN genes showing significant results in a dN/dS analysis. For every identified rapidly evolving (putative positively selected) amino acid site with > 95% confidence, the top-scoring surrounding PFAM domains were summarized in the histogram

Our comparative evolutionary analysis using a dN/dS approach revealed that over 40% of the *H. glycines* core single-copy orthogroups exhibited evidence of rapid evolution (Fig. 4a). Specifically, 1,276 of 3,373 orthogroups had significant dN/dS ratios greater than 1, and 1,137 contained codon sites under diversifying selection as identified by codeml (*p* < 0.01) (Supplementary Tables S10–S11). Protein domain annotation of genes under rapid adaptation identified 1,596 distinct Pfam domains (Supplementary Table S12).

Among the most frequent were the P-loop NTPase (28), protein kinase (16), and GPCR-like 7TM receptors (15) (Fig. 4b). Additional abundant domains included Armadillo-type folds (14), WD40 repeats (15), and various zinc finger families (10). Gene Ontology enrichment analysis further revealed significant overrepresentation of categories related to signal transduction (GO:0007165, FDR = 0.002), chromatin modification (GO:0016568, FDR = 0.007), and ion channel activity (GO:0005216, FDR = 0.013). KEGG pathway analysis highlighted enrichment in pathways associated with environmental information processing, including signal transduction and transcriptional regulation. Together, these results indicate that a substantial portion of the SCN core genome is subject to diversifying selection, with enrichment in domains and pathways linked to cellular signaling, regulatory processes, and protein–protein interactions.

### Variant discovery from the SCN pangenome graph

To characterize large-scale genomic variation, we built a reference pangenome graph incorporating eight chromosome-scale SCN assemblies. We generated a graph representation for each chromosome, capturing both shared and lineage-specific PAVs. This approach identified 9,495 single nucleotide variants (SNVs), 4,567 small InDels (insertions and deletions of length < 50 bp), and 3,344 large PAVs (> 50 bp) across the genomes. Many of these variants were located in genic regions, suggesting potential functional consequences for gene expression and regulation. The graph-based framework provides a comprehensive view of genomic diversity within SCN and serves as a valuable resource for further studies on gene presence-absence variation and evolutionary dynamics.

**Fig. 5:**
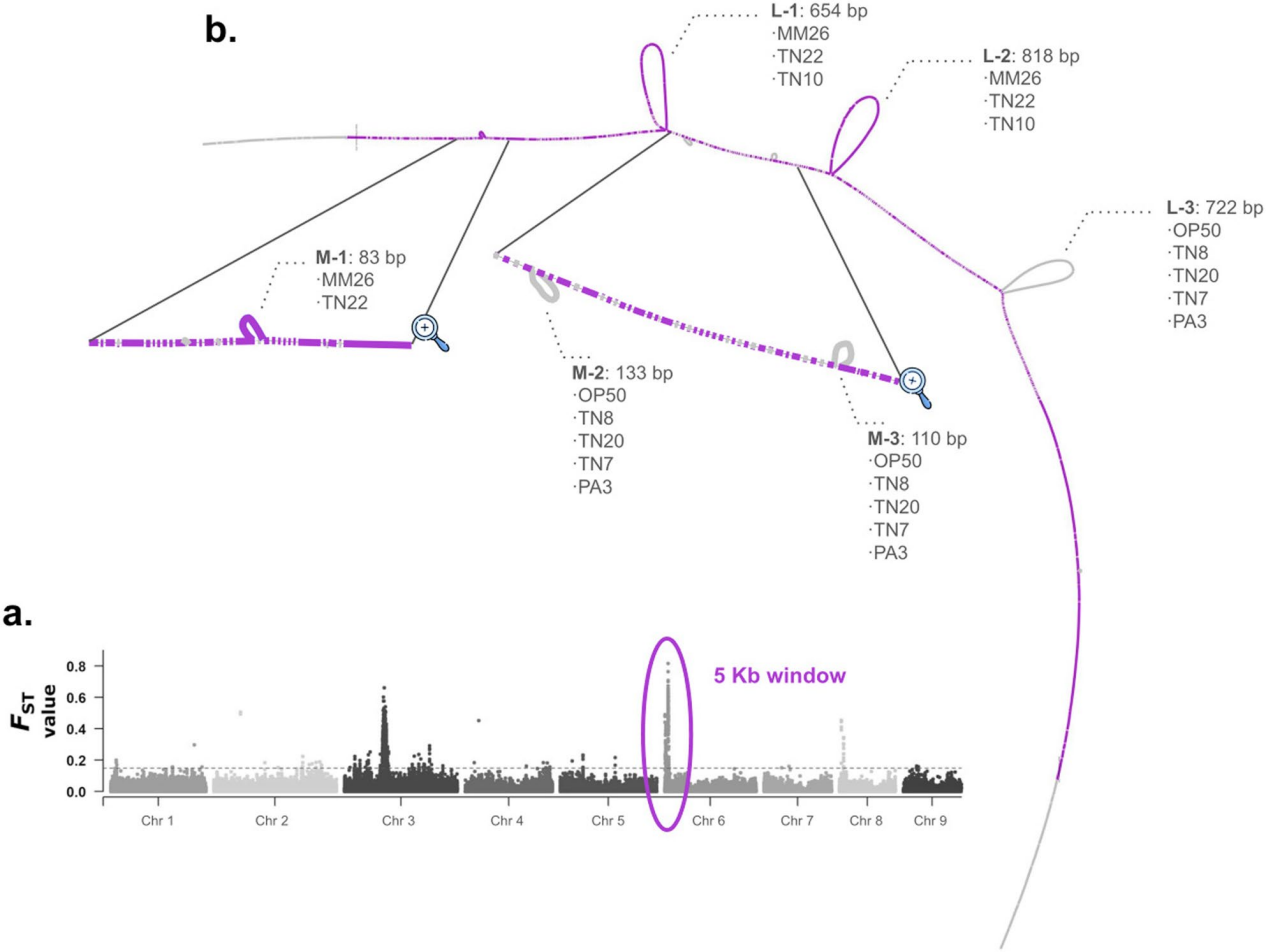
Graph-based analysis of genomic variation in regions of interest (ROIs) within the SCN genome. **a** Identification of ROI from genome-wide selection scans [12], with a strong peak on chromosome 6 highlighted by a 5-kb window (purple). **b** Structural variants (SVs) identified within the chromosome 6 ROI using a pangenome graph. Subgraphs revealed three large SVs (L-) and three smaller SVs (M-)

We investigated the genetic variation within two regions of interest (ROIs) in the SCN genome, focusing on loci previously associated with increased virulence [12]. Specifically, we targeted genomic intervals on chromosomes 3 and 6, where strong signatures of selection were detected. For the chromosome 6 ROI, we extracted a 5-kb window centered around the most significant SNPs and analyzed their local sequence diversity using a graph-based approach (Fig. 5a). The graphs were constructed from seven high-quality de novo assemblies, with TN10 serving as the reference. Subgraphs representing the ROI on chromosome 6 revealed multiple PAVs, including three large (654 bp, 818 bp, and 722 bp) and three smaller (83 bp, 133 bp, and 110 bp) regions of variation, which we refer to as L-1 through L-3 and M-1 through M-3, respectively (Fig. 5b).

Within the chromosome 6 ROI, the PAVs segregated into two major haplotypes. The first haplotype, observed in TN22, TN10, and MM26, consists of the variants M-1, L-1, and L-2. The second haplotype, shared by OP50, TN8, TN20, TN7, and PA3, includes M-2, M-3, and L-3. These contrasting configurations may underlie functional differences in virulence among SCN populations. However, since each genome assembly was generated from pooled individuals, it remains uncertain whether these patterns represent true, individual-level haplotypes. Thus, further validation is required to confirm the biological significance of the structural divergence observed in the graph.

### Variations in secretome genes

Secreted proteins play a central role in determining virulence in SCN and the outcome of the interactions with soybean plants [13, 32]. Based on the prediction of known signal peptides and transmembrane domains we identified a total of 12,678 genes in SCN which potentially encode secreted proteins (Supplementary Table S13). Each genome had an average secretome size of 1,408 genes, with MM26 showing the largest set of secreted genes (1702 genes) and TN7 the smallest (1220 genes).

The variation in secretome size among SCN genomes suggests potential population-specific adaptations, with certain populations possessing a more extensive repertoire of effectors which may influence host infection. While most secreted proteins were shared across all genomes, we also identified a subset of genes that were genome-specific, indicating potential roles in differential virulence or host compatibility. Notably, several secretome genes are located in regions of structural variation, suggesting that genomic rearrangements may contribute to effector diversification.

Clustering the predicted secretomes using OrthoFinder revealed several effector families that varied across HG types. While 35–39% of the secretomes were shared among all populations, only 113 genes were found to be single-copy orthologs. In addition, we identified a small set of race-specific secretome orthogroups (Supplementary Table S14). Race 2, which includes HG type 1.2– populations (MM26, TN22, TN10), contained 31 exclusive orthogroups, whereas Race 4, which includes HG type 1.2.3– populations (TN8, OP50, TN20, X12), contained 97 exclusive orthogroups. No exclusive families were detected for Race 1 (TN7) or Race 3 (PA3), likely due to their single-genome representation. These patterns indicate that virulent HG types harbor distinct effector complements that may contribute to population-specific pathogenicity.

## Discussion

### Genomic Landscape and Population Diversity in SCN

We analyzed the genomes of nine SCN populations spanning a range of HG types, including seven new reference-quality genome assemblies generated in this study. This effort aimed to capture more of the genetic diversity within the species, provide reference genomes for populations differing in HG type, and identify key genes contributing to its biology and adaptive strategies. State-of-the-art long-read sequencing technologies, such as PacBio and Oxford Nanopore Technologies, have demonstrated the ability to resolve repetitive regions and large genomic gaps in complex genomes [33–35]. By integrating Hi-C data, we successfully reconstructed nine pseudomolecules for each genome, aligning with the known chromosome count of the species [30]. The re-annotation of another previously published SCN genome [10] using an ensemble method pinpoints new, uncatalogued genes. The gene space covered in X12 increased from 11,882 genes in the previously reported genome to 22,972 genes in our annotation. This more robust pipeline opens new avenues for the identification of genes of interest in SCN.

SCN is widely known for its ability to rapidly adapt to resistant soybean cultivars, with a large number of HG types that are capable of maintaining high population densities on the currently available sources of resistance [8, 9]. Previous studies have highlighted the challenge of understanding diversity in species with high degrees of genetic diversity and population differentiation [36], as variant calling using single reference assemblies can fail to capture the full spectrum of variation within a species and can lead to errors in quantifying rare alleles in the population [37–39]. Here, we employed a robust comparison of the genetic landscape across multiple distinct SCN populations to provide a more nuanced view of common, shared haplotypes along with the essential genes involved in vital cell functions in SCN, known as the core genome. Nearly 50% of the SCN genome is core (9,887 genes). Core genes are known to be involved in essential functions [40], which align with the fundamental biological processes enriched in the SCN genome, including communication, protein synthesis, and metabolism (Fig. 3 d).

### Gene Content and Structural Variation Underlying SCN Adaptation

Accessory genes, representing nearly half (48%) of the SCN genome and varying across populations or lineages, likely play crucial roles in adaptation and virulence. Such accessory genes are commonly found in microbial pathogens with short generation times and are critical to virulence and environmental adaptation [41]. In SCN specifically, accessory genes are enriched for biological processes such as transport, repair, and translation (Fig. 3 d). The proportion of accessory genes identified in SCN exceeds that reported for several other plant pathogens, including *Dickeya solani* (7%), *Pyrenophora teres* (13%), and *Zymoseptoria tritici* (41%) [40, 42, 43]. This expanded accessory genome likely arises primarily through segmental duplication and horizontal gene transfer events [41], potentially explaining the elevated number of expanded gene families observed in SCN (Fig. 3b). While these comparisons are drawn from microbial and fungal pathogens due to the current lack of published pangenomes in nematode species, they provide a useful context for evaluating genome plasticity and accessory gene content among plant-parasitic species.

The open nature of the SCN pangenome points to extensive genomic plasticity and heterogeneity across populations (Fig. 3c). Such patterns are commonly observed in plant-associated microbes, where frequent presence-absence variation underlie rapid adaptation to host defenses and environmental pressures [28]. The apparent openness of its pangenome highlights the capacity of SCN populations to harbor distinct repertoires of genetic variation, potentially contributing to their broad pathogenic variability and capacity to overcome host resistance. These findings underscore that the current pangenome, while informative, does not yet provide a fully comprehensive view of SCN diversity and that sequencing additional populations will be necessary to capture the full extent of its genomic repertoire.

### Selective Pressures and Evolutionary Dynamics in SCN

Our integrative approach revealed signatures of strong selective pressures acting on the SCN core genome, with an unexpectedly large proportion (38%) of core single-copy genes showing elevated dN/dS. BEB analysis further identified rapidly evolving sites enriched in domains associated with parasitism, host invasion, and immune suppression (Supplementary Table S10; Fig. 4b). This pattern aligns with expectations under the Red Queen hypothesis, in which genes mediating host–pathogen interactions evolve rapidly in response to continual selective pressure [44]. Several of the domains under selection, including P-loop NTPases and GPCR-like receptors, are closely linked to SCN’s ability to perceive soybean rhizosphere cues and regulate effector secretion, providing molecular-level support for this co-evolutionary arms race.

Several studies have demonstrated that recent gene duplication can generate unusually high dN/dS values [84–86]. When a gene duplicates, one copy often experiences relaxed selective pressure or diverges functionally, producing a temporary spike in the rate of amino-acid evolution. In SCN, we observed extensive lineage-specific gene family expansions, revealed by CAFE (Fig. 1b). We suggest that families of paralogous SCN parasitism genes undergo frequent cycles of duplication and loss, creating many single-copy genes with no close extant homologs that have in fact experienced a recent history of paralog divergence. These events leave behind elevated sequence-level divergence that would remain hidden in a single-genome analysis but becomes detectable in a pangenome framework.

Together, our results collectively reveal that SCN harbors a highly dynamic genome shaped by persistent, multi-layered evolutionary pressures. Assuming the elevated dN/dS values reflect gene family turnover, the scale of gene family turnover we observe exceeds the number of duplicated loci reported in earlier SCN genome analysis (around 20%) [22]. Analysis of the pangenome therefore indicates that SCN gene families are even more dynamic than previously recognized. Such rapid cycles of duplication and loss, combined with rapid and relatively unconstrained sequence evolution, could provide a continual source of genetic novelty. Taken with other evidence, it is clear that the SCN population possesses a hyper-dynamic pangenome capable of responding quickly to selective pressures imposed by resistant soybean cultivars and diverse soil environments.

### Haplotype Diversity in Virulence-Associated Regions

The graph-based analysis works as a good framework to resolve structural diversity within regions of interest, particularly in loci associated with virulence. In the chromosome 6 ROI, we identified multiple PAVs that segregate into two distinct putative haplotypes, revealing structural configurations that are not easily captured by linear reference-based analyses. These contrasting haplotypes suggest that large-scale structural variation represents an important layer of genetic diversity shaping host–parasite interactions in SCN.

Such structural differences may alter gene content, regulatory elements, or local chromatin organization, ultimately influencing the expression of virulence-associated genes. The observation of two major haplotypes across populations is consistent with the possibility that selection maintains alternative genomic arrangements in response to diverse host environments. These patterns highlight how PAVs and other forms of structural divergence contribute to the adaptive evolution of virulence-linked regions.

Assembling genomes from pooled individuals limits our ability to phase haplotypes with certainty, underscoring the need for future validation with long-read or single-nematode sequencing. These structural features represent promising targets for future functional and population-level studies. However, because the assemblies represent combined material from multiple individuals, the resulting haplotypes and structural variants may represent population-level composites rather than true individual haplotypes. Once validated, the contrasting haplotypes identified here may serve as informative molecular markers for rapid detection of SCN virulence types, supporting screening efforts for disease-resistant soybean varieties.

## Conclusion

The construction of a graph-based SCN pangenome offers multiple advantages for future research. The availability of multiple high-quality assemblies allows us to map reads more precisely across a diversity of genomic contexts, improving confidence in the detection of structural variation. Variant calling across populations using a pangenome graph reduces the possibility of reference bias and supports a more refined understanding of population structure and evolutionary dynamics in SCN. This has particular relevance for deciphering the genetic basis of virulence, especially when paired with functional insights from genes that are significant in our genome-wide dN/dS analyses.

By comparing multiple populations, we exposed otherwise hidden variation that plays a central role in SCN’s biology, diversity, and adaptive potential. Our analyses revealed a broad set of candidate genes potentially contributing to virulence and host adaptation. These candidate loci, enriched in domains linked to host-pathogen interactions, represent valuable targets for functional validation and future resistance screening efforts. By leveraging the full breadth of SCN genomic diversity, this pangenome resource empowers a more precise and informed approach to understanding the biology of SCN and thus to combating nematode infections in soybean.

Our findings indicate that the SCN core genome is not a static set of conserved genes, but rather a dynamic population reservoir shaped by continual adaptation. The functional categories under selection align with processes critical for host interaction, signal perception, and defense evasion, consistent with ongoing co-evolutionary dynamics between SCN and soybean resistance mechanisms. Taken together, this pangenome provides a robust foundation for identifying virulence determinants and advancing future efforts to develop durable resistance in soybean.

## Methods

### Nematode populations and DNA collection

We sequenced eight SCN populations (MM26, PA3, OP50, TN7, TN8, TN10, TN20, and TN22) in this study. Each population was developed by single cyst descent and mass-inbred for continuous experimental adaptation 12 generations/year by the named nematologists and subsequently maintained by Melissa Mitchum at the University of Missouri from 2004 to 2019, exceeding 10 years of selection (> 120 generations). HG type testing for 7 populations (Supplementary Table S0) was conducted according to the standardized cyst evaluation protocol [24, 25] at the time of egg collection for sequencing. The Female Index (FI), expressed as a percentage, was used as a measure of reproduction on a resistant line, calculated as (mean number of females on the resistant indicator line)/(mean number of females on the susceptible soybean cv. Lee74) x 100 [24]. TN7 typed as HG type 2.5.7 (race 1), TN22 and MM26 typed as HG 1.2.5.7 (race 2), PA3 typed as HG Type 7 (race 3), TN8 and OP50 typed as HG 1.2.3.5.6.7 (race 4), and TN20 typed as HG 1–7 (race 4). TN10 was typed as 1.2.6.7 according to its previous description [11].

Egg samples were collected, surface-sterilized in 0.02% sodium azide solution for 20 min, washed in dH20, and flash-frozen and stored at −80 °C before processing. For TN7, TN8, TN20, TN22, and OP50, egg pellets were ground in liquid nitrogen, lysed with SDS and Proteinase K, and purified using a series of centrifugation, RNAse A treatment, and isopropanol precipitation steps. Additional purification steps were applied to TN7, TN22, and OP50, including column-based extractions and phenol/chloroform treatments. For PA3 and MM26, DNA extraction was outsourced to PolarGenomics LLC (Ithaca, NY), using proprietary protocols for high-molecular-weight (HMW) DNA extraction. The TN10 population was maintained as in previous studies [11, 22]. For X12, we obtained the assembled genome and transcriptomic sequencing reads [10] deposited on DRYAD. These sequences were subsequently used in our study for genome re-annotation, as detailed in the corresponding section of the Methods.

### Whole-genome Sequencing

Illumina paired-end shotgun libraries were prepared for TN7, TN8, TN20, TN22, and OP50 using the Hyper Library Construction Kit (Kapa Biosystems), followed by sequencing on an Illumina HiSeq 4000 platform (151 cycles, paired-end). For long-read sequencing, Oxford Nanopore libraries were generated for TN7, TN8, TN20, TN22, and OP50 using 1D barcoded libraries (SQK-LSK108), loaded on SpotON FLO-MIN-106 (R9.4.1) flowcells for 48 h, and sequenced on a GridIONx5 instrument. For MM26, an ultra-long Oxford Nanopore library was prepared using the XL protocol (SQK-LSK109) and sequenced on a SpotON R10.3 flow cell. Basecalling was performed using different versions of Guppy, depending on the sample.

For PA3, a PacBio Continuous Long Read (CLR) library was generated, where DNA was sheared to 30 kb, adapter-ligated, and sequenced using Sequel V3 chemistry on a PacBio Sequel platform. Additionally, PacBio HiFi libraries were prepared for both PA3 and MM26, where DNA was sheared to 12 kb, converted into SMRTbell libraries, and sequenced on a Sequel II platform with Circular Consensus Sequencing (CCS) mode.

Dovetail Chicago and Hi-C libraries were generated for all SCN HG types. Chicago libraries involved chromatin reconstitution followed by formaldehyde fixation and digestion with DpnII. Hi-C libraries followed a similar approach but included chromatin cross-linking within the nucleus. Both libraries were sequenced on an Illumina HiSeq X platform (2 × 150 nt). These libraries were used for scaffolding and genome assembly enhancement.

### Genome Assembly

Genome assemblies were constructed using sequencing platform-specific methods. For Oxford Nanopore reads, we employed Porechop for adapter trimming and NanoFilt for read filtering, followed by error correction using Canu (v1.7-1.8.8.8) [45–47]. Corrected reads were assembled with SMARTdenovo [48], aligned using minimap2 [49], and polished iteratively with Nanopolish. Illumina paired-end reads were trimmed with Trimmomatic [50] and polished using Pilon for ten iterative rounds [51].

For PA3, PacBio CLR reads were corrected with Canu and assembled with SMARTdenovo, while PacBio HiFi reads were aligned and polished iteratively. MM26 PacBio HiFi reads were also assembled with SMARTdenovo, with ultra-long Oxford Nanopore reads error-corrected via HiCanu, followed by scaffolding using LINKS [52]. For final genome scaffolding, Chicago and Dovetail Hi-C libraries, along with shotgun reads, were utilized within the HiRise pipeline [53], iterating between alignment and gap-closing steps.

The TN10 genome was assembled using both Nanopore and PacBio RSII reads, starting with 20 FLYE assemblies under various parameters [54]. After filtering out non-mapped regions and removing N’s, iterative Quickmerge assembly [55] was performed to finalize the assembly based on BUSCO gene presence. BUSCO genes missing from the assembly were added by mapping BUSCO-containing Nanopore reads and converting them to FASTA format [56]. The assembled contigs were scaffolded with Hi-C data using the Juicer/3D-DNA/Juicebox pipeline, followed by polishing with Nanopore reads using Pilon to correct local misassemblies. Duplicate haplotigs were removed through self-mapping with Minimap2, and any unmapped or small contigs (< 1 kb) were eliminated. The final assembly was polished with PacBio reads using Pilon, followed by additional manual scaffolding with the Hi-C pipeline.

### Gene Prediction and Annotation

RNA-seq reads were aligned to the genome using the STAR v2.7.6 two-pass method for splice junction optimization [57]. Samtools v1.16.1 was used to merge, sort, and convert SAM to BAM files [58]. Nanopore cDNA was aligned to each genome using minimap2 v2.28 with the parameters -x splice -G 2000 [49]. Read alignments were filtered for secondary, supplementary, unmapped reads, and those with alignment scores lower than q20. Both BAM files were assembled into transcripts using StringTie v2.2.1 [59] and annotated into genes using BRAKER v2.1.2 [60]. Portcullis v1.22 was used to identify high-confidence splice junctions [61], and TransDecoder v5.5.0 was used to identify putative proteins in the StringTie v2.2.1 prediction [62]. These gene sets were combined in Mikado v2.3.2 in permissive mode, without filtering transcripts with non-canonical splice junctions [63].

The completeness of each genome was evaluated with BUSCO v6.0.0 [56], using the lineage datasets nematoda_odb12. To characterize genomic repeats, we used RepeatModeler v2.0.2 [64] to build repeat libraries, followed by RepeatMasker v4.1.2-p1 [65] to mask the identified repeats.

To assess the orthology between genes in SCN and outgroups, peptide sequences were compared using OrthoFinder v.2.5.4 [66] to detect Clusters of Orthologous Groups (COGs). We retrieved genomic sequences and annotations of four outgroup species (*H. schachtii*, *G. rostochiensis*, *G. palida*, *C. elegans*) from public databases (Supplementary Table S1). Single-copy COGs were identified based on the longest isoform of each protein-coding gene.

We applied OrthoFinder using three configurations: (a) nine SCN genomes, without outgroups; (b) nine SCN genomes, and *H. schachtii* as the outgroup; and (c) nine SCN genomes, and four outgroup species. Core- and pan-genome sizes were estimated through resampling combinations of 1 to 9 genomes. OrthoVenn3 [67] was utilized to visualize the cluster results of core, soft-core, accessory, and unique genomes. To identify synteny blocks and large structural variations across the SCN genomes, we utilized GENESPACE v0.9.3 [68] along with OrthoFinder v.2.5.4 using default parameters.

### Phylogenetic relationships in SCN

To assess the phylogenetic relationships between related species and the SCN accessions, 803 single-copy COGs were concatenated into a single sequence and aligned using MAFFT [69] with the “-automated1” parameter. TrimAI was used to retain the most reliable positions of the alignment. A Maximum Likelihood tree was generated using FastTree [70] with WAG and GAMMA models, and 1,000 bootstrap replicates. The tree was visualized using MEGA11 software [71]. Gene family expansion and contraction were estimated using CAFE using default parameters [72].

### Analysis of dN/dS rates

To investigate evolutionary rates of single-copy orthologs in SCN, we computed synonymous (dS) and nonsynonymous (dN) substitution rates, using *H. schachtii* as the outgroup (assembly GCA_019095935.1, NCBI). Multiple sequence alignments of the predicted proteins were converted to codon alignments using PAL2NAL for each COG [73].

We used CODEML [74] with parameters ‘model = 0, NSites = 0,1,2’ to estimate the ratio of dN/dS (ω) under multiple site models, using the phylogenetic tree as input. LTR statistics at 1% significance were applied to compare nested site models, initially identifying orthogroups with variability of selective pressure among amino acid sites (M0 vs. M1a), and subsequently identifying sites under predicted potential positive selection (M1a vs. M2a). Orthogroups with significant support for the M2a model were further examined by evaluating their dN/dS ratios. Genes with ω > 1 were classified as potentially under differential selection. For cases where M1a failed to be rejected, dN/dS ratios were also analyzed.

To refine candidate sites under selection, we performed a Bayesian Empirical Bayes (BEB) analysis to identify sites with over 99% confidence of being under selection. Protein domains surrounding these sites were annotated using InterProScan [75] to provide functional context for the differentially selected genes.

### Identification of SVs and pan-reference genome construction

To identify structural variants (SVs), we constructed a reference pangenome graph from eight chromosome-level SCN assemblies using Minigraph-Cactus [76]. A separate graph was constructed for each chromosome. TN10 was defined as the reference assembly for the graph construction. Graph visualization and inspection were performed using Bandage [77], which allowed us to explore structural features such as bubbles and alternative paths. Additionally, we used ODGI [78] to manipulate and query the graph, extract subgraphs corresponding to specific regions of interest, and analyze path-level topology relevant to structural variation.

### Secretome identification

The secretome, defined as the set of secretory proteins, was identified using a three-step prediction pipeline [79] involving SignalP, DeepTMHMM, and TargetP. Initially, SignalP [80] was used with default settings to generate a set of proteins containing signal peptides for each genome. DeepTMHMM [81] was then applied to predict and exclude proteins with transmembrane domains. Finally, TargetP [82] was used to predict and exclude proteins with mitochondrial targeting signals.

**Table 1:** Summary statistics of nine *de Novo* genome assemblies of SCN. The statistics of TN10 (HG 1.2.6.7) correspond to the improved assembly and re-annotation after its latest release [11]. *For X12 (HG 1.2.3.4.5.6.7), all gene-related statistics correspond to our new annotation, and the assembly statistics were extracted from the original publication [10]. BUSCO completeness scores refer to annotated proteins

Assembly	MM26	OP50	PA3	TN10	TN20	TN22	TN7	TN8	X12*
Total assembly size (Mb)	144.93	112.42	123.67	115	117.16	125.74	122.31	119.48	141.35
Number of sequences	1,178	101	404	9	194	88	111	58	267
Longest scaffold (Mb)	18.94	16.17	19.86	18.8	19.44	19.54	20.83	18.47	21.03
Number of scaffolds > 10 Mb	7	7	6	6	6	8	7	6	7
N50 scaffold length (Mb)	14.13	13.5	13.69	12.67	13.01	14.1	14.23	13.68	16.27
Assembly anchored in chromosomes (%)	83.91	98.99	95.55	100	98.24	99.3	99.71	99.61	91.25
Total repeat element ratio (%)	44.49	40.42	41.28	40.71	40.19	41.89	40.5	41.27	42.66
GC content (%)	37.63	37.36	36.87	36.99	36.73	36.64	36.71	36.97	36.89
Number of genes	24,795	16,287	20,765	20,368	20,216	17,797	18,231	17,569	22,972
Mean gene length (bp)	3,363.21	4,220.91	3,560.45	3,256.42	3,365.12	4,398.25	4,157.62	4,171.39	3,311.82
Number of annotated genes	15,121	11,357	12,735	12,803	12,934	11,806	12,167	11,753	14,114
BUSCO Completeness (%)	95.4	95.9	94.5	95.5	96.3	93.8	94	95.8	87.8

## Supplementary Information

Supplementary material 1.

## Data Availability

The genome assemblies and annotation files generated in this study have been deposited in public repositories. Raw sequencing data are available in NCBI under multiple BioProject IDs (Supplementary Table S1). Assemblies and annotations are also available under SCNbase [[83]](https:/www.zotero.org/google-docs/?sdk08X). Additional datasets, including core gene IDs and secretome annotations, are included as Supplementary Files.
